# Recirculation in single lumen cannula venovenous extracorporeal membrane oxygenation: A non-randomized bi-centric trial

**DOI:** 10.3389/fmed.2022.973240

**Published:** 2022-08-31

**Authors:** Christoph Fisser, Oscar Palmér, Marko Sallisalmi, Michael Paulus, Maik Foltan, Alois Philipp, Maximilian V. Malfertheiner, Matthias Lubnow, Thomas Müller, Lars Mikael Broman

**Affiliations:** ^1^Department of Internal Medicine II, University Medical Center Regensburg, Regensburg, Germany; ^2^ECMO Centre Karolinska, Pediatric Perioperative Medicine and Intensive Care, Karolinska University Hospital, Stockholm, Sweden; ^3^Department of Cardiothoracic Surgery, University Medical Center Regensburg, Regensburg, Germany; ^4^Department of Physiology and Pharmacology, Karolinska Institutet, Stockholm, Sweden

**Keywords:** ECMO, recirculation, ultrasound dilution, cannula, configuration, hemolysis, risk factor

## Abstract

**Background:**

Recirculation is a common problem in venovenous (VV) extracorporeal membrane oxygenation (ECMO). The aims of this study were to compare recirculation fraction (R*f*) between femoro-jugular and jugulo-femoral VV ECMO configurations, to identify risk factors for recirculation and to assess the impact on hemolysis.

**Methods:**

Patients in the medical intensive care unit (ICU) at the University Medical Center Regensburg, Germany receiving VV ECMO with femoro-jugular, and jugulo-femoral configuration at the ECMO Center Karolinska, Sweden, were included in this non-randomized prospective study. Total ECMO flow (*Q*_*EC*_), recirculated flow (Q_REC_), and recirculation fraction R*f* = Q_REC_/Q_EC_ were determined using ultrasound dilution technology. Effective ECMO flow (Q_EFF_) was defined as Q_EFF_ = Q_EC_ * (1–R*f*). Demographics, cannula specifics, and markers of hemolysis were assessed. Survival was evaluated at discharge from ICU.

**Results:**

Thirty-seven patients with femoro-jugular configuration underwent 595 single-point measurements and 18 patients with jugulo-femoral configuration 231 measurements. R*f* was lower with femoro-jugular compared to jugulo-femoral configuration [5 (0, 11) vs. 19 (13, 28) %, respectively (*p* < 0.001)], resulting in similar Q_EFF_ [2.80 (2.21, 3.39) vs. 2.79 (2.39, 3.08) L/min (*p* = 0.225)] despite lower Q_EC_ with femoro-jugular configuration compared to jugulo-femoral [3.01 (2.40, 3.70) vs. 3.57 (3.05, 4.06) L/min, respectively (*p* < 0.001)]. In multivariate regression analysis, the type of configuration, distance between the two cannula tips, ECMO flow, and heart rate were significantly associated with Rf [B (95% CI): 25.8 (17.6, 33.8), p < 0.001; 960.4 (960.7, 960.1), *p* = 0.009; 4.2 (2.5, 5.9), p < 0.001; 960.1 (960.2, 0.0), p = 0.027]. Hemolysis was similar in subjects with Rf > 8 vs. ≤ 8%. Explorative data on survival showed comparable results in the femoro-jugular and the jugulo-femoral group (81 vs. 72%, *p* = 0.455).

**Conclusion:**

VV ECMO with femoro-jugular configuration caused less recirculation. Further risk factors for higher R*f* were shorter distance between the two cannula tips, higher ECMO flow, and lower heart rate. R*f* did not affect hemolysis.

## Background

Venovenous extracorporeal membrane oxygenation (VV ECMO) is a method of providing patients with oxygenated blood in the case of severe respiratory failure (1, 2). In VV ECMO, deoxygenated blood is drained from the venous compartment, oxygenated by a membrane lung (ML), and subsequently returned to the venous compartment. Recirculation is evident, when returned fully oxygenated blood is aspirated into the drainage cannula without adding any contribution to systemic oxygenation (3). Recirculation is undesirable because it diminishes the effectiveness of ECMO support and may thus compromise systemic oxygenation.

Peripheral VV ECMO including the jugular vein offers two different configuration options for cannulation with two single lumen cannulae: femoro-jugular and jugulo-femoral. Another peripheral configuration is femoro-femoral. It should be noted that the first part of these terms denotes the site of the drainage cannula and the latter part the site of the return cannula (4). In the femoro-jugular configuration, the tip of the drainage cannula is positioned in the upper inferior vena cava (IVC). In the jugulo-femoral configuration, the tip of the drainage cannula is placed into the right atrium (RA). In both types of configurations, the return cannula is accordingly placed in a large vein on the opposite side of the diaphragm. Placement of the drainage cannula close to the RA as in the jugulo-femoral configuration may result in higher recirculation fraction (R*f*) than achieved with the femoro-jugular configuration.

Both ECMO blood flow (Q_EC_) and recirculated flow (Q_REC_) can be measured. The R*f* is defined as R*f* = Q_REC_/Q_EC_, and effective ECMO flow (Q_EFF_) can be calculated as Q_EFF_ = Q_EC_ * (1–R*f*) (5, 6). In the literature, R*f* values range from 2 to 60%, and may depend on various factors such as Q_EC_, the type of cannula, and the drainage site (6–13). Limited oxygen delivery during VV ECMO can be partly compensated by increasing Q_EC_, usually at the expense of increasing R*f*. Such increase, however, may expose blood to increased shear forces and the associated risk of hemolysis (14).

Thus, the aims of this study were to investigate the difference in R*f* between the femoro-jugular and the jugulo-femoral configuration, to identify risk factors for R*f* , and to assess the impact on hemolysis.

## Methods

### Trial design

This non-randomized investigator-initiated bi-centric prospective study compared the R*f* of the femoro-jugular to that of the jugulo-femoral configuration in VV ECMO. The study protocol was reviewed and approved by the local institutional Ethics Committees (Ethical review number: Stockholm: 2014/945-31; Regensburg: 17-737-101). Written informed consent was obtained from all patients. The study was conducted according to the Declaration of Helsinki on Good Clinical Practice.

### Study subjects

The study included adult patients (>18 years of age) treated with VV ECMO for severe respiratory failure [PaO_2_/FiO_2_ < 85 mmHg or refractory respiratory acidosis with pH < 7.25 on optimized positive end-expiratory pressure (PEEP)] at the University Medical Center Regensburg, Germany, between January 2018 to January 2021 or at the ECMO Center Karolinska, Karolinska University Hospital, Stockholm, Sweden, between April 2018 to May 2019. The difference in the two inclusion periods was related to technical malfunction of the measuring device. In addition, patients from a previous study at the ECMO Center Karolinska were considered eligible for the Stockholm cohort due to slow recruitment and failure of measurement probes with delayed delivery in accordance with the ethical committee (6).

**Figure 1:**
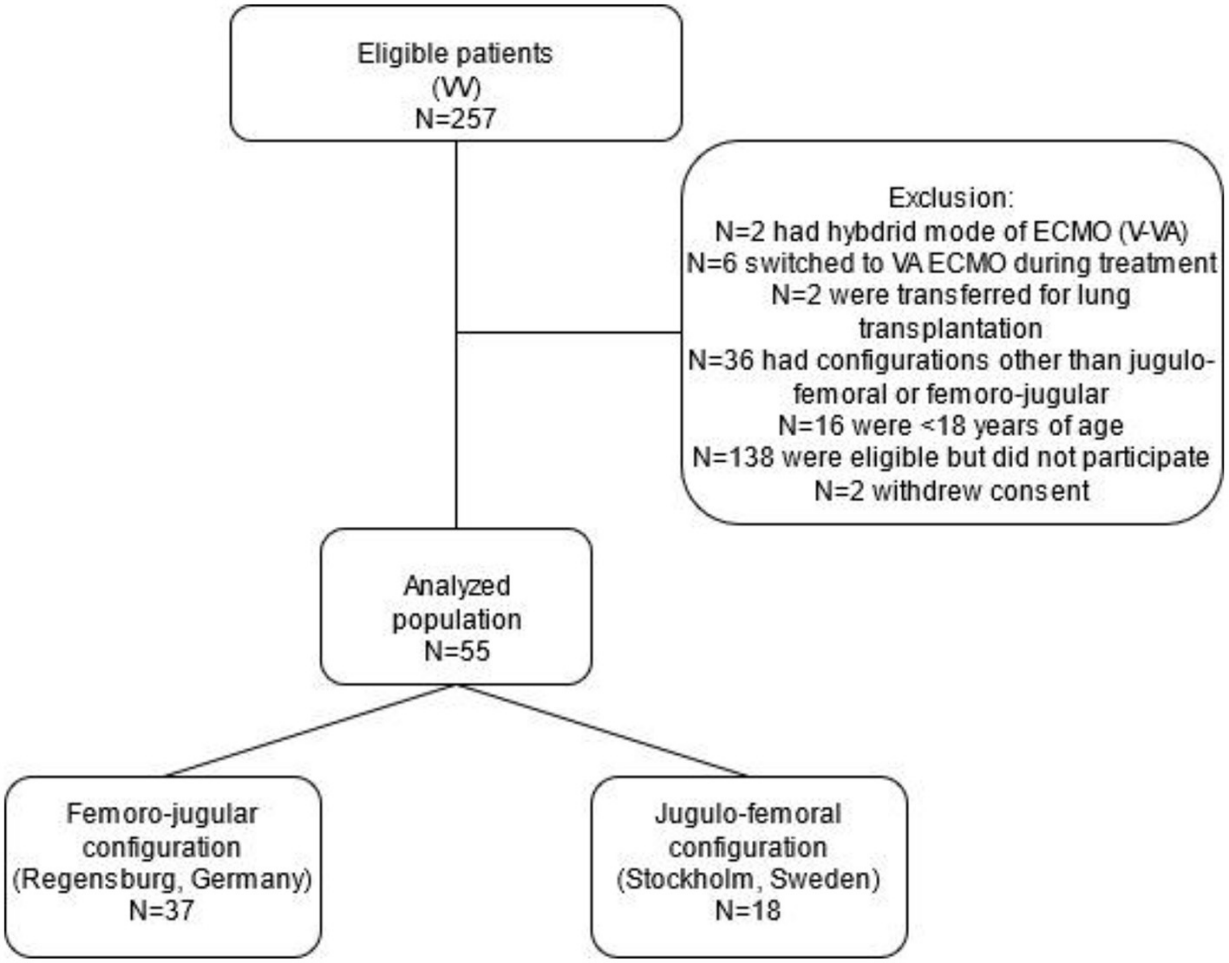
Flowchart of the prospective non-randomized bi-centric study comparing the femoro-jugular to the jugulo-femoral configuration in venovenous extracorporeal membrane oxygenation in terms of recirculation. ECMO, extracorporeal membrane oxygenation; VA, venoarterial; VV, venovenous.

Exclusion criteria were VV ECMO configuration other than jugulo-femoral or femoro-jugular configuration, venoarterial, venopulmonary, or any hybrid mode of ECMO, age <18 years, expected survival of <48 h, and pronounced hemodynamical instability (Figure 1).

Besides demographics, other criteria to be analyzed were cannula specifics (diameter and length), the tip-to-tip distance between the cannulae, ECMO specifics [Q_EFF_, saturation pre-ML (S_PRE_O_2_)], hemolysis [plasma free hemoglobin (fHb) >500 mg/L measured by a commercial available calorimetric assay, C462-0A Catachem, Oxford, CT, USA (14), or HemoCue Plasma/Low Hb, HemoCue, Ängelholm, Sweden], vasoactive inotropic score [dopamine dose (μg/kg/min) + dobutamine (μg/kg/min) + 100 × epinephrine dose (μg/kg/min) + 50 × levosimendan dose (μg/kg/min) + 10 × milrinone dose (μg/kg/min) + 10,000 × vasopressin (units/kg/min) + 100 × norepinephrine dose (μg/kg/min) (15)] ventilation parameters, daily assessment of net fluid balance and survival at discharge from intensive care unit (ICU). Details of ventilation management is presented in the supplement. Cardiac output was measured by means of echocardiography.

### Trial procedures and recirculation fraction

In Regensburg, the tip of the femoral drainage cannula was positioned in the IVC, and the jugular return cannula was placed into the superior caval vein (femoro-jugular configuration). The aim was a tip-to-tip distance of ≥15 cm to minimize potential recirculation. After cannulation, the tip position was verified by sonography or radiographic imaging. In general, 21 French (Fr) cannula, 38 cm length was used as drainage and 19 Fr/15 cm as return cannula (HLS, Getinge Cardiovascular, Rastatt, Germany).

In Stockholm, the tip of the jugular drainage cannula was positioned in the RA, and correct placement was confirmed by echocardiography or radiographic imaging. The tip of the femoral return cannula was placed in the iliac vein (jugulo-femoral configuration). By default, the drainage cannula was 25 Fr/38 cm (HLS, Getinge Cardiovascular) and for return 19 Fr/18 cm (Bio-Medicus, Medtronic, Tolochenaz, Switzerland).

In both centers, adaptions were allowed according to the treating physician. Further details for both centers on cannulation strategy have been previously published (6, 16). The distance between the cannula tips was assessed by means of computed tomography or chest X-ray. Further details are presented in the supplements. Recirculation fraction was measured in supine position using ultrasound dilution technology (UDT), (ELSA^®^, Transonic Systems Inc., Ithaca, NY, USA) as described previously (5, 6). Measurements were allowed any time during ECMO therapy if the patient was hemodynamically stable. One ultrasonic flow probe was applied to the drainage tube in proximity to the patient, the other transducer was placed in proximity to the return cannula. A rapid (<3 s) bolus of 20 mL room tempered saline was injected into the ECMO circuit before the ML. The probes measured ultrasound velocity in the blood and the blood flow rate by means of the Doppler technique. The respective ultrasound velocity data was processed with the ELSA device. The quotient of the drainage to the return curve areas was considered the R*f*. At least two measurements were taken to account for any variability due to breathing efforts. If large differences were observed, further measurements were undertaken, flawed values deleted and the mean of at least two measurements, regarded as valid taken, to chart. For each measurement, Q_EC_, R*f*, and Q_EFF_ were recorded. Q_EC_ was increased or decreased in steps of 300–500 mL/min. The magnitude and number of respective flow rates in each session depended on the patient status and the prevailing Q_EC_. After assessment, the Q_EC_ was returned to the clinical baseline setting, and the aggregated saline volume used was added to the daily fluid balance. Vital and ventilatory parameters were recorded with each measurement.

The reproducibility of paired UDT measurements has been reported to differ by 5.6% in children and possibly even less in adults with a greater distance between the two cannula tips and to be similar to other methods using thermodilution and lithium indicator methods (17, 18).

### Statistics

Descriptive statistics are presented as numbers (n), range, and fractions (%), and continuous data as median [interquartile range (IQR): 25%; 75%], as appropriate. Continuous data were compared with the Mann-Whitney *U*-test, and categorical data with the Chi^2^ test. A multivariate linear regression model was calculated, including all independent variables with *p* < 0.1 in the univariate model. Multivariate linear regression analyses were conducted to identify risk factors for R*f*, including known possible risk factors such as ECMO and cannula specifics as well as hemodynamic and respiratory parameters as published previously (13). Linear quadratic regression models were used to assess associations between the R*f* , Q_EC_, and Q_EFF_. A two-sided *p* < 0.05 was considered statistically significant. Data entry and calculation were done with Microsoft EXCEL365 ProPlus (Microsoft, USA) and IBM SPSS Statistic software version 25.0 (SPSS Inc. Chicago, IL, USA).

## Results

### Study population

Fifty-five patients were prospectively enrolled in this bi-centric study, 37 received VV ECMO with the femoro-jugular configuration (Regensburg, Germany), and 18 VV ECMO with the jugulo-femoral configuration (Stockholm, Sweden) (Figure 1). Patient characteristics were similar between the two groups (Table 1), except for higher PaO_2_/FiO_2_ ratios, higher doses of norepinephrine and lower bilirubin levels in the femoro-jugular than in the jugulo-femoral group. The most frequent diagnoses at admission to the ICU were bacterial (36%) and viral pneumonia (36%). Median support on ECMO was 17 (9, 26) days in the femoro-jugular group and 13 (8, 22) days in the jugulo-femoral group (*p* = 0.468).

**Table 1:** Patient characteristics and parameters at the time of decision for extracorporeal membrane oxygenation.

**Variables**	***n***	**Femoro-Jugular configuration**	***n***	**Jugulo-Femoral configuration**	***p*-value**
Age, years	37	57 [46, 68]	18	55 [44, 66]	0.795
Sex, male	37	24 (65%)	18	11 (61%)	0.786
BMI, kg/m^2^	37	27.7 [24.7, 34.6]	13	27.0 [24.5, 33.8]	0.732
PaO_2_/FiO_2_ ratio, mmHg	36	84 [68, 107]	16	50 [45, 66]	**<0.001**
SOFA	37	10 [9, 13]	18	10 [8, 11]	0.363
Norepinephrine, μg/kg/min	33	0.17 [0.07, 0.33]	8	0.0 [0.00, 0.08]	**0.006**
pH before ECMO	36	7.25 [7.16, 7.33]	18	7.28 [7.20, 7.37]	0.279
Lactate before ECMO, mg/dL	37	13 [10, 19]	18	16 [6, 20]	1.000
Days in hospital before ECMO	37	2 [1, 10]	17	7 [3, 10]	0.384
Days on mechanical ventilation before ECMO	37	1 [0, 3]	17	3 [1, 8]	0.135
Days on RRT before ECMO	22	0 [0, 0]	14	0 [0, 5]	0.077
Bicarbonate, mmol/L	35	24.9 [21.4, 28.0]	13	23.0 [21.4, 30.7]	0.991
CRP, mg/L	37	237 [96, 283]	9	283 [40, 411]	0.567
White blood cells, 10^9^/L	37	12.9 [8.1, 19.8]	17	14.0 [2.4, 21.1]	0.473
Platelets, 10^9^/L	36	227 [138, 326]	17	213 [162, 250]	0.804
Bilirubin, mg/dL	37	0.6 [0.5, 1.1]	17	1.9 [1.2, 5.5]	**<0.001**
Creatinine, mg/dL	37	1.2 [0.8, 2.0]	18	1.3 [1.0, 1.6]	0.647

BMI, body mass index; CRP, C-reactive protein; ECMO, extracorporeal membrane oxygenation; FiO_2_, fraction inspired oxygen; PaO_2_, arterial partial pressure of oxygen; RRT, renal replacement therapy; SOFA, sequential organ failure assessment. Significant p < 0.05 are marked in bold.

### Cannulae

Significantly smaller drainage and return cannulae were used in the femoro-jugular than in the jugulo-femoral group [21 (21, 23) Fr vs. 25 (25, 25) Fr, *p* < 0.001; 18 (17, 19) Fr vs. 19 (19, 20) Fr, *p* = 0.003, Supplementary Table 1]. The drainage cannula was located below the diaphragm in patients receiving the femoro-jugular configuration and above the diaphragm in patients with the jugulo-femoral configuration [−8.3 (−10.0, −4.0) cm vs. 5.5 (4.7, 8.1) cm, *p* < 0.001]. The tip-to-tip distance between the two cannulae was less in the femoro-jugular configuration [19 (17, 21) cm vs. 36 (33, 40) cm, *p* < 0.001] than for jugulo-femoral subjects.

### Recirculation fraction

**Figure 2:**
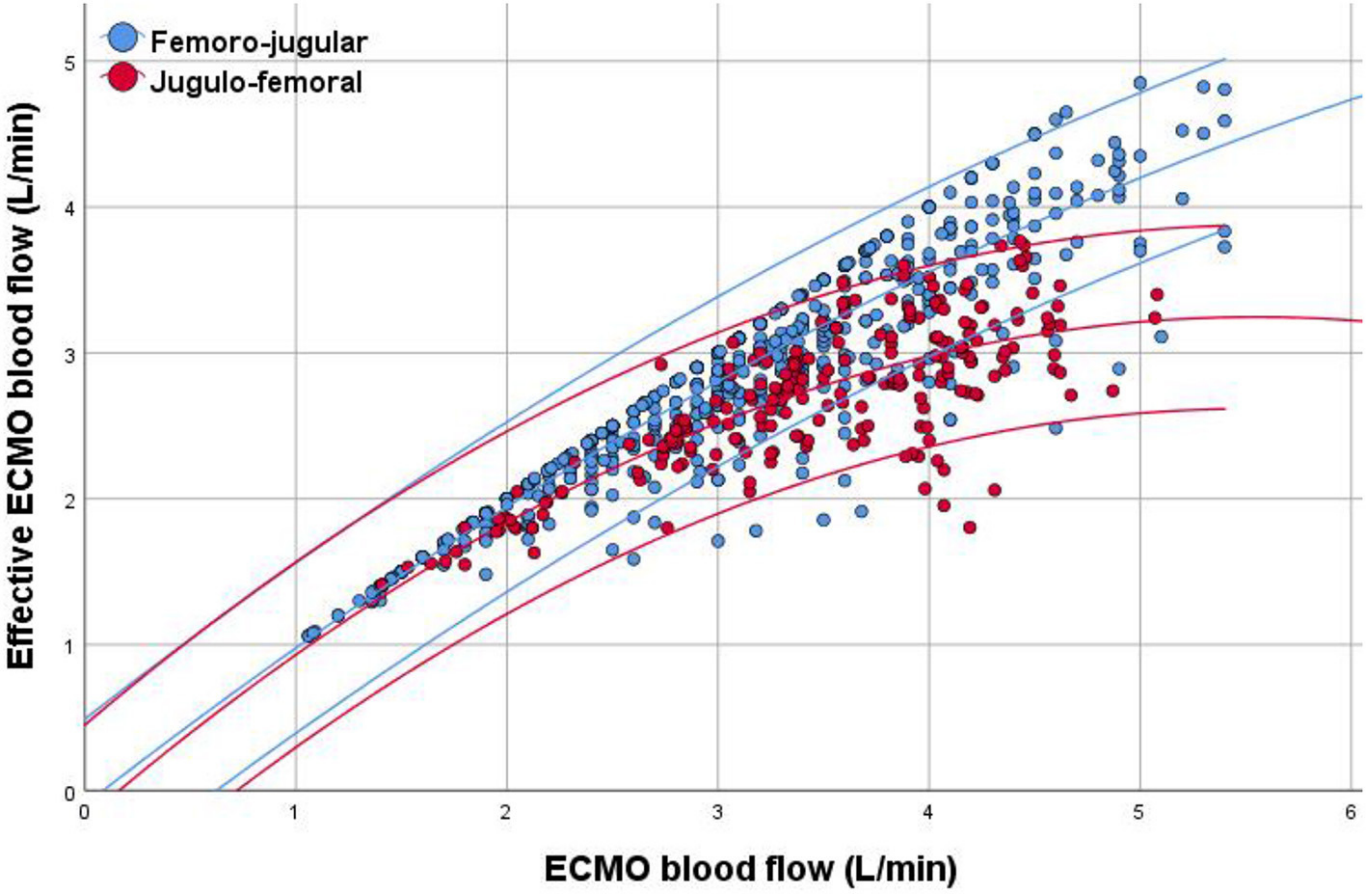
Scatter plot of extracorporeal membrane oxygenation (ECMO) blood flow and effective ECMO blood flow. Data are expressed as quadratic regression analysis with a line fitted to the mean and the 95% confidence intervals according to ECMO configuration, demonstrating lower recirculation fraction in the femoro-jugular configuration than in the jugulo-femoral configuration group.

We conducted 826 single-point measurements of recirculation, 595 in the femoro-jugular and 231 in the jugulo-femoral configuration group. Median R*f* of all measurements was 9 [0, 17] %. Extracorporeal flow was lower in the femoro-jugular configuration [3.01 (2.40, 3.70) vs. 3.57 (3.05, 4.06) L/min, *p* < 0.001, Figure 2]. However, since R*f* was significantly lower in femoro-jugular than in jugulo-femoral group [5 (0, 11) vs. 19 (13, 28) %, *p* < 0.001], extracorporeal support in terms of Q_EFF_ was similar between both groups [2.80 (2.21, 3.39) vs. 2.79 (2.39, 3.08) L/min, *p* = 0.225], respectively. Further configuration related data is depicted in Table 2.

**Table 2:** Characteristics in association with recirculation according to configuration per single point measurement.

**Variables**	**Femoro-Jugular configuration**	**Jugulo-Femoral configuration**	***p*-value**
Distance between the two cannulae tips, cm	19 [17, 21]	36 [33, 40]	**<0.001**
ECMO flow, L/min	3.0 [2.4, 3.7]	3.6 [3.0, 4.1]	**<0.001**
Recirculation fraction, %	5 [0, 11]	19 [13, 28]	**<0.001**
Effective ECMO flow, L/min	2.8 [2.2, 3.4]	2.8 [2.4, 3.1]	0.225
Aspartate aminotransferase, U/L	51 [34, 85]	75 [48, 116]	**0.002**
Alanine aminotransferase, U/L	44 [32, 69]	63 [40, 84]	0.061
Lactate dehydrogenase, U/L	378 [296, 502]	432 [345, 612]	**0.019**
Plasma free hemoglobin, mg/L	36 [28, 48]	35 [28, 85]	0.987
Mean arterial pressure, mmHg	72 [64, 78]	71 [66, 76]	0.595
Heart rate, /min	85 [70, 102]	96 [84, 106]	**<0.001**
Cardiac output, L/min	6.1 [5.4, 7.5]	5.7 [4.6, 7.7]	0.426
SaO_2_, %	96 [94, 97]	93 [90, 99]	**<0.001**
Saturation pre membrane lung, %	68 [62, 74]	75 [72, 78]	**<0.001**
F_i_O_2_ (ventilator), %	45 [40, 55]	60 [50, 60]	**<0.001**
Peak inspiratory pressure, cmH_2_O	22 [20, 26]	26 [19, 28]	**0.022**
Positive end-expiratory pressure, cmH_2_O	11 [8, 15]	8 [5, 10]	**<0.001**
Respiratory rate, /min	13 [10, 16]	20 [15, 25]	**<0.001**
Tidal volume, mL	277 [205, 398]	574 [385, 743]	**<0.001**

Data are expressed as median [25th percentile, 75th percentile]. F_i_O_2_, fractional inspired oxygen set via ventilator. Significant p < 0.05 are marked in bold.

Furthermore, higher measurements of Rf were seen in patients with higher Q_EC_, larger drainage and larger return cannulae, lower heart rate, and more intense mechanical ventilation [i.e., higher peak inspiratory pressure, higher tidal volume and higher respiratory rate (Supplementary Tables 2, 3)].

In univariate analysis, R*f* was associated with Q_EC_, ECMO configuration, distance between the two cannula tips, mean arterial pressure, heart rate, positive end-expiratory pressure, respiratory rate, tidal volume, size of drainage, and size of return cannulae (Table 3). Data were robust in multivariate analysis for higher R*f* , which was associated with ECMO configuration (jugulo-femoral approach), higher Q_EC_, shorter distance between the two cannula tips, and lower heart rate (Table 3). Sensitivity analysis in those with spontaneous breathing yielded comparable results to the overall group, except for ventilatory parameters (Supplementary Table 4). However, in a further sensitivity analysis including only those with measurement of tip-to-tip distance by means of computed tomography or only those with plain X ray measurement, R*f* was only associated with the tip-to-tip distance in the univariate but not in the multivariate analysis (Supplementary Table 5).

**Table 3:** Univariate and multivariate linear regression of recirculation fraction.

	**Univariate analysis**		**Multivariate analysis**	
	**B (95% CI)**	***p*-value**	**B (95% CI)**	***p*-value**
Configuration (Center)	13.7 (12.3, 15.2)	**<0.001**	25.8 (17.6, 33.9)	**<0.001**
Distance between the two cannula tips, cm	0.3 (0.2, 0.5)	**<0.001**	−0.4 (−0.7, −0.1)	**0.009**
ECMO flow, L/min	6.3 (5.5, 7.0)	**<0.001**	4.2 (2.5, 5.9)	**<0.001**
Mean arterial pressure, mmHg	−0.1 (−0.2, 0.0)	**0.046**	0.0 (−0.1, 0.1)	0.667
Heart rate, /min	0.1 (0.0, 0.1)	**0.017**	−0.1 (−0.2, 0.0)	**0.026**
Cardiac output, L/min	−1.3 (−2.8, 0.3)	0.106		
Vasoactive inotropic score	0.0 (0.0, 0.0)	0.206		
FiO_2_, %	5.5 (−1.5, 12.4)	0.126		
Positive inspiratory pressure, cmH_2_O	0.1 (−0.1, 0.3)	0.327		
Positive end-expiratory pressure, cmH_2_O	−0.5 (−0.7, −0.3)	**<0.001**	−0.2 (−0.5, 0.2)	0.334
Respiratory rate, /min	0.4 (0.2, 0.6)	**<0.001**	−0.2 (−0.4, 0.0)	0.096
Tidal volume, mL	0.013 (0.007, 0.018)	**<0.001**	−0.006 (−0.014, 0.002)	0.126
Drainage cannula, Fr	3.1 (2.7, 3.5)	**<0.001**	−0.9 (−2.5, 0.7)	0.255
Return cannula, Fr	1.6 (1.0, 2.1)	**<0.001**	−0.5 (−1.5, 0.5)	0.352

ECMO, extracorporeal membrane oxygenation; FiO_2_, fraction inspired oxygen. Significant p-values < 0.05 are marked in bold.

### Hemolysis and fluid balance

**Figure 3:**
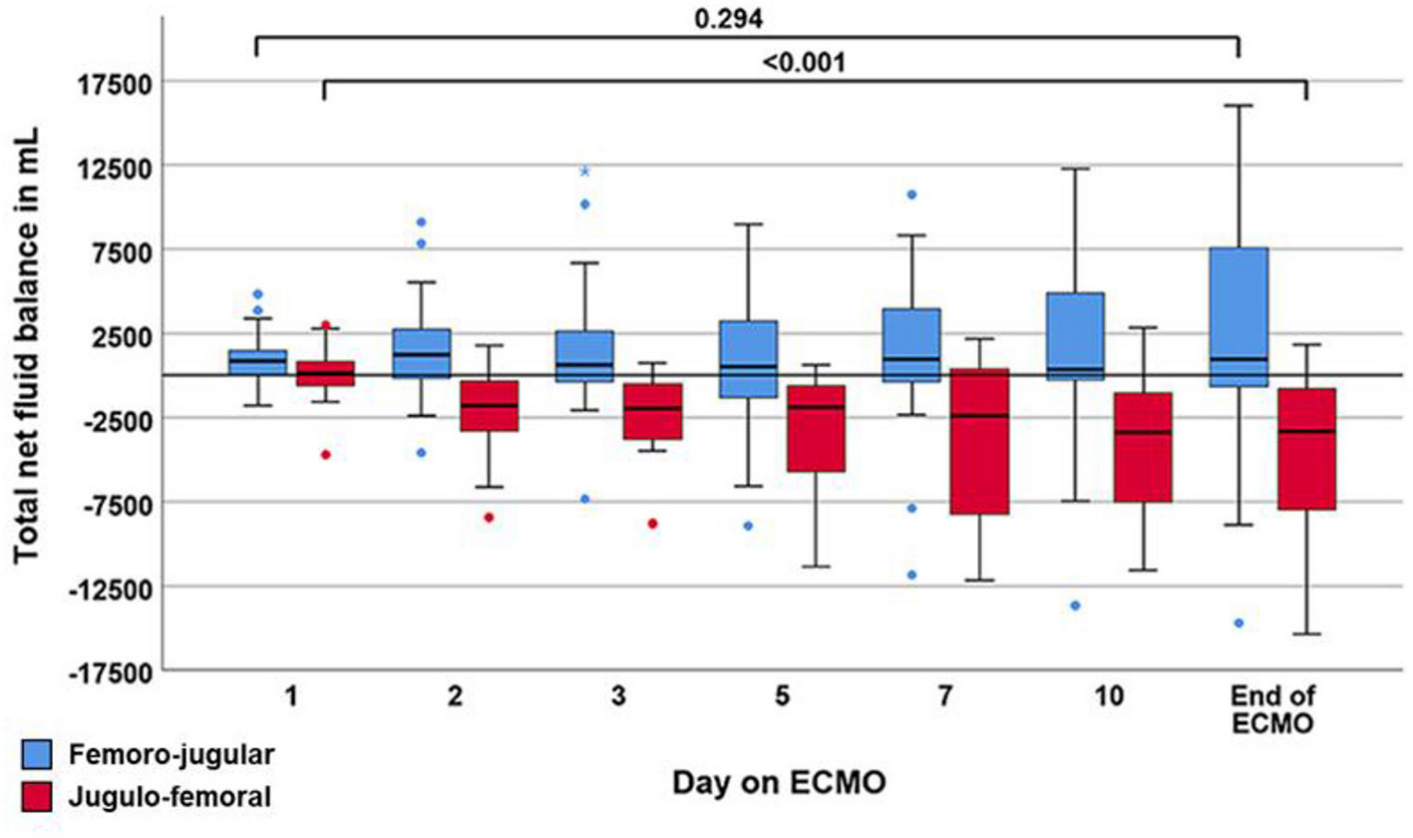
Boxplot showing the trajectory of net fluid balance during the course of extracorporeal membrane oxygenation (ECMO). Data are expressed as median, minimum, maximum, 25th percentile, and 75th percentile. Circles and stars represent outliers with more than one and a half times or more than three times the length of the box from either end of the box.

The fHb neither differed between the two types of configurations nor was it related to a R*f* below or above 9% (Table 2, Supplementary Table 3). Negative pre inlet pump pressures were neither different between groups [femoro-jugular: −5 (−23, 5) vs. jugulo-femoral: −10 (– 33, 8), *p* = 0.363] nor associated with fHB (Supplementary Table 6). Total net fluid balance was similar between the two groups on day one of ECMO therapy but differed between configurations from day two of ECMO therapy onwards (Figure 3, Supplementary Table 7).

## Discussion

This prospective bi-centric study investigated the impact of the flow direction in VV ECMO on R*f* by means of the femoro-jugular and the jugulo-femoral configuration. The femoro-jugular configuration was superior regarding lower R*f* values because it provided a higher Q_EFF_ at similar Q_EC_ compared to the jugulo-femoral configuration. Factors associated with a high R*f* in multivariate linear analysis were jugulo-femoral configuration, shorter distance between the two cannula tips, higher Q_EC_, and lower heart rate. The R*f* did not affect hemolysis.

To our knowledge, this is the first study to systematically compare R*f* in two different peripheral VV ECMO configurations in a clinical context. In general, the R*f* in our study was lower than the values published previously (13), eventually due to lower applied ECMO blood flows in comparison to other ECMO centers using ECMO flows of 5-6 l/min. In particular high ECMO flows were associated with higher R*f* (Figure 2). However, ECMO flows of >5 l/min were rarely applied in this study. Moreover, as R*f* and Q_EFF_ were assessed unnecessary high flows were avoided to reduce mechanical blood trauma. The jugulo-femoral configuration has the predestined disadvantage of draining oxygenated blood more easily because the drainage cannula in the RA is placed amidst blood streaming toward the tricuspid valve, even though the design of a multi-staged cannula may partly reduce this effect (6). It is still unknown if recirculation is a limiting factor for jugulo-femoral configuration in relation to hemolysis, morbidity, and mortality in comparison to femoro-jugular configuration.

In 1998, Rich et al. (19) compared the jugulo-femoral to the femoro-jugular configuration, each with two 23 Fr/25 cm cannulae, in nine patients and found higher maximal Q_EC_ with femoro-jugular configuration; R*f* , however, was not assessed. The study was conducted with neuromuscular blockade in the first day of VV support and with a low frequency (6 min^−1^) inversed ratio pressure control ventilation strategy that may impact central venous volume distribution. Consequently, several centers switched from the jugulo-femoral to the femoro-jugular configuration to be able to provide higher Q_EC_. Today, the femoro-jugular configuration is the more commonly used technique (20).

Traditionally, Q_EC_ is adjusted to meet the patient's need for oxygen, whereas removal of carbon dioxide is adjusted by the amount of sweep gas flowing through the ML. Without R*f* measurement, Q_EFF_ will remain unknown, and only indirect signs such as saturation pre-membrane lung or pulmonary arterial oxygen saturation can be used to evaluate this variable (21). In this study, R*f* measurements showed similar Q_EFF_ in both groups, but adequate ECMO support could be provided more effectively, i.e., with a lower Q_EC_, in the femoro-jugular group.

As R*f* increases, Q_EFF_ may approach a plateau. Beyond an inflection point, arterial oxygen delivery may decrease without the physician noticing it. In our study, recirculation became apparent at higher flows in both configurations, but less pronounced in the femoro-jugular configuration, as supposed to in a simulation model (22). In the same model (22), flow direction and cannula diameter were calculated to have a moderate influence on R*f*. In our multivariate analysis, the size of the cannulae was not associated with R*f* , but jugulo-femoral configuration showed a strong association with higher R*f*, in fact, stronger than any other investigated parameters. Comparable to a computational model, further risk factors with rather modest absolute effects on R*f* were the distance between the two cannula tips and heart rate (22). The higher R*f* with shorter tip-to-tip distance may be reasonable from a pathophysiological perspective because the drainage cannula may easier drain oxygenated blood from the return cannula. In 2018, Togo et al. (23) showed comparable results in an experimental setting in four goats; in their study, the tip-to-tip distance in femoro-jugular configuration was associated with the R*f* as assessed with the limited oxygen saturation calculation method. In an experimental model it was recently shown that a distance of nine to twelve cannula diameters was required for the mix of native venous and ECMO blood to become homogenous (24). Further investigation is necessary with respect to the association of higher Rf with lower heart rate.

### Hemolysis and fluid balance

Hemolysis has been described to be associated with blood flow velocity (14). Therefore, the femoro-jugular configuration with lower R*f* compared to the jugulo-femoral configuration may cause less blood trauma. In this analysis, however, neither the R*f* nor the two configurations did affect hemolysis, maybe due to the appropriate choice of cannula size for the applied blood flow (22).

Beside hemolysis, fluid balance might be affected by the type of configuration. In particular, in femoro-jugular configuration chattering of the tubing (relative hypovolemia; drainage problems from the IVC) is regarded by some to be a more obvious problem than with jugulo-femoral configuration and thus might explain the differences in fluid states. Similar to a recent retrospective study in 27 patients (25), the results of the current study pointed in the same directions, however, due to differences between the groups in patients' characteristics and ECMO management, e.g., timing of ECMO therapy during course of disease or hemodynamic impairment prior to cannulation, these results have to be considered with caution. However, in a computational fluid dynamic model, the recirculation fraction was very constant across different volume states (22).

This finding of easier and faster accomplishment of negative fluid balance using jugulo-femoral configuration, is hypothesis generating and needs further investigation set in the context of the impact of fluid overload and risk of increased mortality (26, 27).

### Limitations

This prospective non-randomized bi-centric study has several limitations restricting the generalizability of its results. The study design was non-randomized and did therefore not account for any possible differences in baseline characteristics, such as requirement of norepinephrine or the number of days on mechanical ventilation before ECMO, which might have particularly affected fluid management. In addition, patients from a previous study at the ECMO Center Karolinska were considered eligible for the Stockholm cohort. The study was conducted by staff with considerable expertise and experience in the use of ECMO, but the general clinical guidelines regarding the care of critically ill patients in the two study centers were neither harmonized nor scrutinized by the study supervisors prior to commencing the study. This may especially be true for the management of volume status. Changes in intrathoracic pressure and venous return due to spontaneous breathing effort may have affected R*f*. The used cannula brands and designs differed between the two centers. ECMO flows >5 l/min were rarely applied. Future studies on R*f* should assess the effect of prone position, intrathoracic and intraabdominal pressures. Patients with femoro-femoral configuration were not evaluated.

## Conclusions

VV ECMO with the femoro-jugular configuration results in less recirculation and thus provides equally effective ECMO support as VV ECMO with the jugulo-femoral configuration but at a lower Q_EC_. Risk factors for higher R*f* were shorter distance between the two cannula tips, higher ECMO flow, and lower heart rate. R*f* did not affect hemolysis. Further studies on the impact of recirculation during VV ECMO are warranted that should include other configurations such as bi-femoral and dual-lumen cannulation.

## Data availability statement

The original contributions presented in the study are included in the article/Supplementary material, further inquiries can be directed to the corresponding author/s.

## Ethics statement

The studies involving human participants were reviewed and approved by the local institutional Ethics Committees (Ethical review number: Stockholm: 2014/945-31; Regensburg: 17-737-101). The patients/participants provided their written informed consent to participate in this study.

## Author contributions

LB was responsible for the study design and the conception. CF and TM co-designed the concept. LB, CF, and TM were responsible for the hypothesis, delineation, the design of the study as well as for the acquisition of data, the analysis and interpretation of this information, for writing the article, and its revision prior to submission. LB, OP, MS, and CF were responsible for drafting the manuscript were involved in the acquisition of data, the analysis and interpretation of this information, and the critical revision of the article prior to submission. MP, MF, AP, MM, ML, and TM were involved in the acquisition of data, the analysis and interpretation of results, and the critical revision of the article prior to submission. LB and CF are the guarantor of the content of the manuscript. All authors read and approved the final manuscript.

## Conflict of interest

MM and LB are advisory board members of the Eurosets Srl., Medolla, Italy. LB is an advisory board member of the Xenios AG, Heilbronn, Germany. The remaining authors declare that the research was conducted in the absence of any commercial or financial relationships that could be construed as a potential conflict of interest.

## Publisher's note

All claims expressed in this article are solely those of the authors and do not necessarily represent those of their affiliated organizations, or those of the publisher, the editors and the reviewers. Any product that may be evaluated in this article, or claim that may be made by its manufacturer, is not guaranteed or endorsed by the publisher.

## Abbreviations

ECMO: extracorporeal membrane oxygenation
fHb: plasma free hemoglobin
IQR: interquartile range
IVC: inferior vena cava
ML: membrane lung
PEEP: positive end-expiratory pressure
PaO_2_/FiO_2_: partial arterial oxygen pressure to fraction of inspired oxygen
Q_EC_: ECMO blood flow
Q_EFF_: effective ECMO flow
Q_REC_: recirculation flow
RA: right atrium
R*f*: recirculation fraction
S_PRE_O_2_: saturation pre membrane lung
VV: venovenous.
