# Corrigendum: Recirculation in single lumen cannula venovenous extracorporeal membrane oxygenation: A non-randomized bi-centric trial

**DOI:** 10.3389/fmed.2022.1045207

**Published:** 2022-10-04

**Authors:** Christoph Fisser, Oscar Palmér, Marko Sallisalmi, Michael Paulus, Maik Foltan, Alois Philipp, Maximilian V. Malfertheiner, Matthias Lubnow, Thomas Müller, Lars Mikael Broman

**Affiliations:** ^1^Department of Internal Medicine II, University Medical Center Regensburg, Regensburg, Germany; ^2^ECMO Centre Karolinska, Pediatric Perioperative Medicine and Intensive Care, Karolinska University Hospital, Stockholm, Sweden; ^3^Department of Cardiothoracic Surgery, University Medical Center Regensburg, Regensburg, Germany; ^4^Department of Physiology and Pharmacology, Karolinska Institutet, Stockholm, Sweden

**Keywords:** ECMO, recirculation, ultrasound dilution, cannula, configuration, hemolysis, risk factor

In the published article, there was an error in Figure 3 as published. The assignment of the colors of the squares to the ECMO configuration was displayed as blue square = “Jugulo-femoral” and red = “Femoro-jugular.” The colors should be blue square = “Femoro-jugular” and red square = “Jugulo-femoral.”

**Figure 3:**
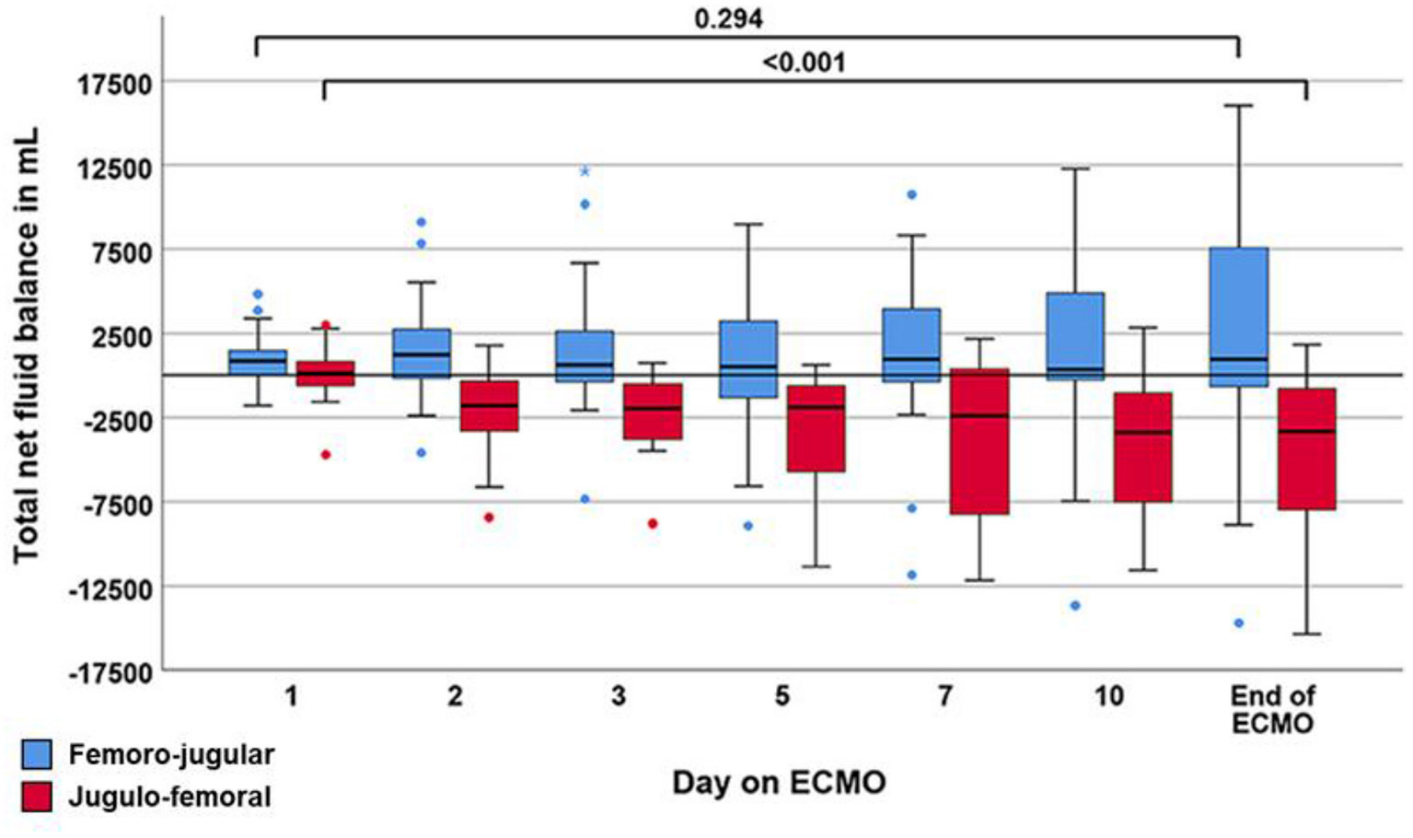
Boxplot showing the trajectory of net fluid balance during the course of extracorporeal membrane oxygenation (ECMO). Data are expressed as median, minimum, maximum, 25th percentile, and 75th percentile. Circles and stars represent outliers with more than one and a half times or more than three times the length of the box from either end of the box.

The corrected Figure 3 appears below.

The authors apologize for this error and state that this does not change the scientific conclusions of the article in any way. The original article has been updated.

## Publisher's note

All claims expressed in this article are solely those of the authors and do not necessarily represent those of their affiliated organizations, or those of the publisher, the editors and the reviewers. Any product that may be evaluated in this article, or claim that may be made by its manufacturer, is not guaranteed or endorsed by the publisher.

