# Supplementary material for: Recirculation in single lumen cannula venovenous extracorporeal membrane oxygenation: A non-randomized bi-centric trial
Source: Front Med (Lausanne). 2022 Aug 31;9:973240. doi: 10.3389/fmed.2022.973240 (PMC9470851; doi:10.3389/fmed.2022.973240)
Supplement: Supplementary file 1 [file Data_Sheet_1.pdf]

Supplemental material:

This appendix has been provided by the authors to give readers additional information about the study.

Supplement to:

Recirculation in single lumen cannula venovenous extracorporeal membrane oxygenation: a non-randomized bi-centric trial

Christoph Fisser[1], Oscar Palmér[2], Marko Sallisalmi[2], Michael Paulus[1], Maik Foltan[3], Alois Philipp[3], Maximilian V. Malfertheiner[1], Matthias Lubnow[1], Thomas Müller[1], Lars Mikael Broman[2,4]

[1)] Department of Internal Medicine II, University Medical Center Regensburg, Regensburg, Germany

[2)] ECMO Centre Karolinska, Pediatric Perioperative Medicine and Intensive Care, Karolinska University Hospital, Stockholm, Sweden

[3)] Department of Cardiothoracic Surgery, University Medical Center Regensburg, Regensburg, Germany

[4)] Department of Physiology and Pharmacology, Karolinska Institutet, Stockholm, Sweden

Corresponding author:
Christoph Fisser, MD
Department of Internal Medicine II
University Medical Center Regensburg
Franz-Josef-Strauss-Allee 11
93053 Regensburg
Germany
Phone: ++49 941 944 17359
Facsimile: ++49 941 944 7365
Email: christoph.fisser@ukr.de

Ventilatory management:

Regensburg: After the initiation of veno-venous extracorporeal membrane oxygenation (VV ECMO) ventilatory settings are decreased to result in a peak pressure ≤30 cmH_2O, a driving pressure ≤14 cm H_2O, most commonly <8 cm H_2O and respiratory rate <12/min. The positive end-expiratory pressure (PEEP) is adjusted individually. In the initial first days controlled ventilation is applied to the patient. The FdO_2 (fractional delivered oxygen in the sweep gas) is kept by 100% during the complete ECMO support. Once the underlying disease was clinically controlled, the ECMO blood and sweep flow are stepwise reduced. In parallel, we allow spontaneous ventilation if no objective or subjective signs of distress are presented. ECMO weaning trials are initiated if the patient is hemodynamically (heart rate<120/min, systolic blood pressure 90-160 mmHg, norepinephrine <0.2 μg/(kg*min) and respiratory ($SpO_2 \geq 90\%$ with $FiO_2 \leq 0.4$ or PaO_2/FiO_2>150 mmHg, pH=7.35-7.45, positive end-expiratory pressure ≤10 cmH_2O, driving pressure<15 cmH_2O, respiratory rate<30/min, tidal volume per kg predicted bodyweight>5 ml/kg, ECMO blood flow≈1.5 l/min, sweep gas flow≈1 l/min) in a stable condition.

Stockholm: After support has been initiated the common ventilator mode would be pressure control and pressures reduced to keep peak pressure <30 cmH_2O and PEEP usually between 8 and 12 cmH_2O depending on patient's initial settings. FiO_2 is reduced to 0.6 from start and then further, usually to 0.3-0.4 the next days. The patient will be scheduled for a tracheostomy within the next one or two days to ease subsequent awakening. The weaning process starts very early on by hourly assessments of pre-membrane and arterial patient blood gases. As the lung recovers patient $paCO_2$ is kept stable within a pre-set range decided during the morning rounds (usually $paCO_2$ 38-60 mmHg). When sweep gas flow is down on 2 L/min (adult) an incremental fraction of carbon dioxide of 0.5% up to a physiologic concentration 5% ($FdCO_2$

0.5-5%) is added to the sweep gas flow (FdO2 1.0) with the continued improvement of ventilatory function. Pre and post-membrane blood gases are evaluated and when the difference between PpreCO2 and PpostCO2 is ≤3 mmHg and the FiO2 is acceptable the patient is ready for a trial-off, i.e. the sweep gas is turned off.

Cannula measurements:

The tip-to-tip measurements were made from plain chest XR (magnification factor due to diverging Röntgen beams by cannula diameter used as reference) or CT scan. A direct line was drawn from the mid of the one tip to the mid of the opposite tip. Measurements were registered in cm with 1 decimal place. In both centers these evaluations were made by experienced ECMO specialist physicians.

Table S1: Cannula specifics according to extracorporeal membrane oxygenation configuration

Variables*	Femoro-jugular configuration N=37	Jugulo-femoral configuration N=18	p-value
Drainage cannula, French			**<0.001**
19	1 (3%)	0 (0%)	
21	25 (68%)	0 (0%)	
23	11 (30%)	0 (0%)	
25	0 (0%)	18 (100%)	
Drainage cannula length, cm			-
38	37 (100%)	18 (100%)	
Return cannula, French			**0.003**
15	3 (8%)	0 (0%)	
17	16 (43%)	1 (6%)	
19	16 (43%)	13 (72%)	
21	2 (5%)	4 (22%)	
Return cannula length, cm			**<0.001**
15	33 (89%)	0 (0%)	
18	0 (0%)	18 (100%)	
23	4 (11%)	0 (0%)	

Data are expressed as n (%). The brand of the cannulas used in the femoro-jugular configuration was HLS (Getinge Cardiovascular, Rastatt, Germany). In the jugulo-femoral configuration, the brand of the drainage cannula was HLS (Getinge Cardiovascular) and Bio-Medicus (Medtronic, Tolochenaz, Switzerland) for the return cannula. *values are rounded. ECMO. Significant p-values ($p < 0.05$) are marked in bold.

Table S2: Cannula specifics according to median recirculation fraction per single point measurement

Variables*	Recirculation ≤8%<br>N=412	Recirculation >8%<br>N=407	p-value
Drainage cannula, French			**<0.001**
19	6 (2%)	1 (0.2%)	
21	312 (76%)	161 (40%)	
23	72 (18%)	41 (10%)	
25	22 (5%)	204 (50%)	
Drainage cannula length, cm			-
38	412 (100%)	407 (100%)	
Return cannula, French			**<0.001**
15	37 (9%)	17 (4%)	
17	165 (40%)	86 (21%)	
19	186 (45%)	267 (66%)	
21	24 (6%)	37 (9%)	
Return cannula length, cm			**<0.001**
15	349 (85%)	159 (39%)	
18	22 (5%)	204 (50%)	
23	41 (10%)	44 (11%)	

Data are expressed as n (%). *values are rounded. Significant p-values (p<0.05) are marked in bold.

Table S3: Characteristics according to median recirculation fraction per single point measurement

Variables	n	Recirculation <9%	n	Recirculation ≥9%	p-value
Configuration (Center)					**<0.001**
Femoro-jugular		390 (95%)		22 (5%)	
Jugulo-femoral		203 (50%)		204 (50%)	
Distance between the two cannulae tips, cm	400	20 [17; 22]	283	19 [17; 31]	0.317
ECMO flow, L/min	411	2.7 [2.1; 3.4]	405	3.6 [3.0; 4.1]	**<0.001**
Recirculation fraction, %	411	0 [0; 5]	405	17 [12; 26]	**<0.001**
Effective ECMO flow, L/min	411	2.7 [2.1; 3.3]	405	2.8 [2.4; 3.2]	**0.015**
Plasma free hemoglobin, mg/L	151	36 [28; 50]	51	35 [28; 46]	0.834
Aspartate aminotransferase, U/L	152	54 [40; 108]	77	57 [36; 83]	0.559
Alanine aminotransferase, U/L	155	44 [31; 78]	82	51 [36; 75]	0.186
Lactate dehydrogenase, U/L	156	380 [296; 493]	82	420 [312; 602]	0.055
Mean arterial pressure, mmHg	180	72 [64; 79]	227	70 [65; 76]	0.240
Heart rate, /min	170	87 [72; 102]	227	95 [81; 106]	**<0.001**
Cardiac output, L/min[a]	44	6.1 [5.4; 7.9]	22	5.5 [4.5; 7.3]	0.145
SaO2, %	176	96 [94; 98]	205	94 [90; 98]	**<0.001**
Saturation pre membrane lung, %	352	69 [63; 75]	290	70 [63; 75]	0.357
FiO2, %	344	45 [40; 55]	227	50 [40; 60]	**0.010**
Positive inspiratory pressure, cmH_2O	312	22 [19; 26]	205	23 [21; 26]	**0.032**
Positive end-expiratory pressure, cmH_2O	334	11 [8; 15]	241	10 [8; 12]	**0.008**
Respiratory rate, /min	146	14 [11; 18]	106	16 [14; 23]	**<0.001**
Tidal volume, mL	156	338 [239; 485]	156	475 [244; 677]	**<0.001**

Abbreviations: ECMO, extracorporeal membrane oxygenation; FiO_2, fraction inspired oxygen; SaO_2, arterial hemoglobin oxygen saturation. [a]measured by echocardiography. p-values (p<0.05) are marked in bold.

Table S4: Univariate linear regression of recirculation in patients with spontaneous breathing

N=176	Univariate analysis	
	B (95% CI)	p-value
Configuration (Center)	10.9 (6.2; 15.6)	**<0.001**
Distance between the two cannula tips, cm	0.3 (0.1; 0.5)	**<0.001**
ECMO flow, L/min	5.5 (4.1; 7.0)	**<0.001**
Mean arterial pressure, mmHg	-0.3 (-0.2; 0.1)	0.724
Heart rate, /min	0.2 (0.0; 0.3)	**0.007**
Cardiac output, L/min	-0.9 (-6.9; 5.1)	0.681
FiO2, %	-8.9 (-20.0; 2.3)	0.118
Positive inspiratory pressure, cmH_2O	0.6 (0.3; 0.9)	**<0.001**
Positive end-expiratory pressure, cmH_2O	0.5 (0.2; 0.8)	**0.003**
Respiratory rate, /min	1.3 (-0.1; 0.4)	0.227
Tidal volume, mL	-0.0 (-0.1; 0.0)	0.769
Drainage cannula, Fr	2.0 (1.0; 3.1)	**<0.001**
Return cannula, Fr	-0.4 (-1.4; 0.6)	0.416

Abbreviations: ECMO, extracorporeal membrane oxygenation; FiO_2, fraction inspired oxygen.
Significant p-values ($p<0.05$) are marked in bold.

Table S5: Uni- and multivariate linear regression of recirculation fraction according to included measurement of distance between the two cannula tips.

	Univariate analysis		Multivariate analysis	
	B (95% CI)	p-value	B (95% CI)	p-value
First model: CT measurement				
Distance between the two cannula tips, cm	0.2 (0.1; 0.4)	<0.001	-0.3 (-0.6; 0.1)	0.109
Second model: Plain X ray measurement				
Distance between the two cannula tips, cm	0.9 (0.5; 1.3)	<0.001	-0.8 (-2.2; 0.7)	0.294

In the first model only those patients with measurement of distance between the two cannula tips by means of computed tomography (CT) were included. In the second model only those measured by plain X ray were included. Both models adjusted for configuration (Center), distance between the two cannula tips, ECMO flow, mean arterial pressure, heart rate, positive end-expiratory pressure, respiratory rate, tidal volume, drainage cannula, return cannula.

Table S6: Univariate linear regression of plasma free hemoglobin according to recirculation fraction

	Univariate analysis	
	B (95% CI)	p-value
Pre inlet pump pressure, mmHg		
Overall group	-0.1 (-0.6; 0.4)	0.638
Femoro-jugular group	0.1 (-0.3; 0.4)	0.684
Jugulo-femoral group	-0.2 (-1.6; 1.2)	0.736

Table S7 Cumulative net fluid balance

Variable	n	Femoro-jugular configuration	n	Jugulo-femoral configuration	p-value
Day 1, mL	37	840 [5; 1885]	16	152 [-689; 855]	0.083
Day 2, mL	37	1236 [-301; 2961]	15	-1816 [-3583; -129]	**<0.001**
Day 3, mL	37	627 [-578; 2788]	15	-1942 [-4460; -300]	**<0.001**
Day 5, mL	36	527 [-1432; 3257]	13	-1910 [-7038; -504]	**0.002**
Day 7, mL	33	945 [-421; 4836]	12	-2423 [-8615; 515]	**0.003**
Day 10, mL	30	361 [-394; 5105]	9	-3406 [-8430; 712]	**0.004**
Day ECMO end, mL	37	965 [-712; 7843]	16	-3322 [-8452; -642]	**<0.001**
Net fluid balance per days on ECMO, mL/day	29	100 [-86; 550]	10	-258 [-857; -43]	**0.002**

Data are expressed as n (%), median [25th percentile; 75th percentile]. Significant p-values ($p < 0.05$) are marked in bold.

Table S8: Ventilatory settings according to spontaneous and controlled breathing

	Spontaneous breathing	Controlled ventilation
FiO2, %	45 [35; 50]	45 [40; 50]
Positive inspiratory pressure, cmH_2O	21 [20; 25]	23 [20; 26]
Positive end-expiratory pressure, cmH_2O	8 [6; 12]	12 [10; 16]
Respiratory rate, /min	16 [12; 26]	12 [10; 15]
Tidal volume, mL	380 [251; 565]	260 [190; 356]

Data are expressed as median [25th percentile; 75th percentile].
